# miRNAs as Key Players in the Management of Cutaneous Melanoma

**DOI:** 10.3390/cells9020415

**Published:** 2020-02-11

**Authors:** Celeste Lorusso, Simona De Summa, Rosamaria Pinto, Katia Danza, Stefania Tommasi

**Affiliations:** Molecular Diagnostics and Pharmacogenetics Unit, IRCCS-Istituto Tumori “Giovanni Paolo II”, 70124 Bari, Italy; lorusso.celeste590@gmail.com (C.L.); s.desumma@oncologico.bari.it (S.D.S.); rosamaria.pinto@hotmail.it (R.P.); danzakatia@gmail.com (K.D.)

**Keywords:** microRNA, melanoma, target therapy, immunotherapy, microenvironment

## Abstract

The number of treatment options for melanoma patients has grown in the past few years, leading to considerable improvements in both overall and progression-free survival. Targeted therapies and immune checkpoint inhibitors have opened a new era in the management of melanoma patients. Despite the clinical advances, further research efforts are needed to identify other “druggable” targets and new biomarkers to improve the stratification of melanoma patients who could really benefit from targeted and immunotherapies. To this end, many studies have focused on the role of microRNAs (miRNAs) that are small non-coding RNAs (18-25 nucleotides in length), which post-transcriptionally regulate the expression of their targets. In cancer, they can behave either as oncogenes or oncosuppressive genes and play a central role in many intracellular pathways involved in proliferation and invasion. Given their modulating activity on the transcriptional landscape, their biological role is under investigation to study resistance mechanisms. They are able to mediate the communication between tumor cells and their microenvironment and regulate tumor immunity through direct regulation of the genes involved in immune activation or suppression. To date, a very promising miRNA-based strategy is to use them as prognosis and diagnosis biomarkers both as cell-free miRNAs and extracellular-vesicle miRNAs. However, miRNAs have a complex role since they target different genes in different cellular conditions. Thus, the ultimate aim of studies has been to recapitulate their role in melanoma in biological networks that account for miRNA/gene expression and mutational state. In this review, we will provide an overview of current scientific knowledge regarding the oncogenic or oncosuppressive role of miRNAs in melanoma and their use as biomarkers, with respect to approved therapies for melanoma treatment.

## 1. Introduction

Cutaneous melanoma is one of the most aggressive types of cancer worldwide, with an incidence that has been increasing over the past 50 years [1].

It occurs from the malignant transformation of melanocytes induced mainly by exposure to intense and prolonged ultraviolet radiation. Unfortunately, this disease can progress rapidly to the advanced aggressive metastatic stage. Depending on the stage and the dissemination of the tumor, different therapeutic options are relied on, such as surgical resection, chemotherapy, radiotherapy, immunotherapy, or targeted therapy. During the past decade, systemic treatment for melanoma has enormously changed as knowledge of the key driver mutations and pathways of tumor cells have led to the development of new therapeutic options. Targeted molecular therapies against specific mutations [2,3] and systemic immunotherapies including immune checkpoint inhibitors [4,5,6], have recently emerged, replacing conventional chemotherapy [7].Notwithstanding the success of the new therapeutic approaches, many research groups have been working to enhance our knowledge of melanoma biology and to identify further reliable biomarkers. Recently, many studies have focused on microRNAs (miRNAs) as important factors in the development, metastasis, and prognosis of melanoma. miRNAs are short (20–25 nucleotides) and single-stranded non-coding RNAs, which target the 3′-untranslated region of an estimated 30% of all human genes at its 5′end domain (position 2 to 8), called the “seed region,” and thus play a role as oncogenes or oncosuppressors [8]. They regulate gene expression at the post-transcriptional and translational levels through degradation of mRNA or a translation block. This review discusses the state of the art of studies on miRNAs and their role in response to therapies, their relationship with other “druggable” pathways, and their potential use as clinical biomarkers.

## 2. State of the Art of Targeted Therapy Options

Novel treatments for cutaneous metastatic melanoma (CMM) have been proposed based on the central role of the mitogen activated-protein kinase (MAPK) pathway in this disease. The MAPK pathway is activated by extracellular binding of receptor tyrosine kinases (RTK), leading to the activation of the rat sarcoma (RAS) family protein, which subsequently activates intracellular serine-threonine protein kinases of the rapidly accelerated fibrosarcoma (RAF) family (ARAF, BRAF, CRAF). RAF activation triggers the phosphorylation of MAPK extracellular receptor kinase (MEK), which in turn, phosphorylates extracellular signal-regulated kinase (ERK). ERK regulates both cytosolic targets and nuclear transcription factors, thus leading to cellular proliferation, cellular differentiation, survival, and apoptosis. Activated ERK also provides negative feedback at various levels of the pathway [9]. BRAF mutations are more frequent in melanomas that develop in sun-exposed skin. About 50% of CMM harbor an activating mutation of the BRAF gene that consists in the substitution of a single nucleotide in codon 600. The most common mutation is the result of a substitution of glutamic acid for valine in codon 600 (BRAF^V600E^), which occurs in approximately 90% of BRAF-mutant melanomas. The second most common mutation is BRAF^V600K^ (substituting valine for lysine), which accounts for 5%–6% of BRAF-mutant melanomas [10].

Different clinical characteristics (i.e., gender, age) have been reported between patients with *BRAF* p.V600E and p.V600K mutation. Vemurafenib and Dabrafenib are selective oral BRAF inhibitors (BRAFi) that have been licensed by the Food and Drug Administration (FDA, Hampton, VA, USA) for the treatment of unresectable or metastatic melanomas harboring activating BRAF^V600^ mutations [2,11,12]. The initial efficacy of BRAFi is followed by multiple resistance mechanisms caused by inter-tumor (differences between primary and metastatic tumors) or intra-tumor (differences in features of subclones within a tumor) heterogeneity. These mechanisms usually depend on the recovery of the MAPK pathway or the activation of other pathways such as the PI3K/AKT/mTOR pathway through IGF1R and PDGFRb upregulation [13,14].

For this reason, a valid strategy is to target downstream signaling effectors like MEK 1/2. Cobimetinib and Trametinib are oral selective MEK inhibitors (MEKi) approved by the FDA for the treatment of unresectable or metastatic melanomas [11,12]. Clinical trials have shown that BRAFi/MEKi combination therapy improves survival compared to single-agent treatment [15].

MEKi has proven to be effective also in NRAS-mutant melanomas [16]. NRAS and BRAF mutations are usually mutually exclusive in melanoma. The most common RAS mutations occur in codons 12, 61, or 13; 15% of cases have point mutations. RAS proteins function as small GTPases with low intrinsic catalytic activity. Cycling of the RAS protein between a guanosine-5’-triphosphate (GTP)-bound active state and a guanosine diphosphate (GDP)-bound inactive state is catalyzed, respectively, by guanine nucleotide exchange factors (GEFs) and GTPase activating proteins (GAPs). Mutations in NRAS favor the formation of GTP-bound, activating RAS proteins. One of the approaches to inhibit RAS activation has been to directly target RAS by competing for its GTP binding, similar to kinase inhibitors, which compete with ATP.

However, to date, direct pharmacological inhibition of mutant RAS proteins is difficult because of their very tight binding to GDP/GTP [17]. No therapy has yet been approved for NRAS-mutant melanoma. Two MEKi, Binimetinib, and Pimasertib, have been shown to improve progression-free survival (PFS) vs. Dacarbazine significantly but have not proved to provide an overall survival (OS) benefit. Double inhibition with MEKi in combination with PI3Ki or PI3K/mTORi or AKT inhibitors has been used in clinical trials.

Unfortunately, such a combination was too toxic to allow adequate dosing for an antitumor effect, thus leaving a lack of targeted approaches for *NRAS*-driven melanomas [18]. There are no treatment options available for wildtype-BRAF/NRAS melanomas, which constitute ~30% of all CMMs. Another potentially actionable target gene is c-KIT, a tyrosine-protein kinase that encodes for a receptor essential for the proliferation and survival of mature melanocytes. KIT is often altered in mucosal malignant melanomas, where it activates intracellular signaling cascades, including the MAPK, PI3K, and JAK-STAT pathways. The most common c-KIT mutations in melanoma are L676P and K642E [19]. Trials conducted with Imatinib mesylate, an inhibitor of KIT and other RTKs, in patients with *c-KIT*–mutant melanoma, have reported median times to disease progression of approximately 3 months that are significantly lower than the time to progression when Imatinib is used to treat gastrointestinal stromal tumors (GIST) (median time to progression of 18 months). Despite the presence of the same mutation, it is unclear why there is such a difference in response between KIT-mutant melanoma and GIST, suggesting that there may be other pathways involved in this treatment resistance [19]. The neurofibromin 1 (NF1) gene is considered one of the driver genes in melanomas, specifically in chronically sun-exposed or older subjects and in desmoplastic melanoma. It encodes for a GTPase-activating protein that inhibits the activity of RAS proteins and negatively regulates MAPK signaling. NF1 mutations are present in <15% of melanoma cases and may be present together with NRAS/BRAF mutations. Combination therapy with MEKi and mTORi has been observed to produce an antitumor effect in *BRAF/NF1*-mutant allografts [20].

### 2.1. miRNAs Acting as Oncosuppressors in Melanoma

**Figure 1:**
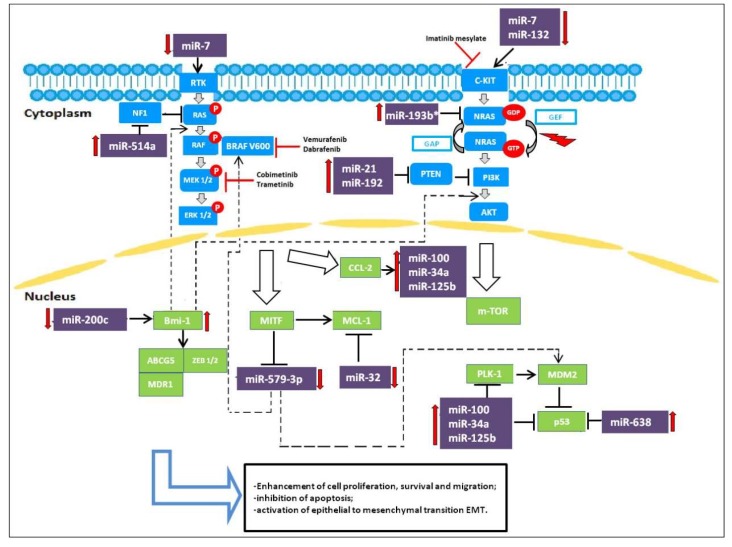
miRNAs involved as oncogenes and oncosuppressors targeting MAPK and PI3K/AKT pathways in melanoma resistant cells to BRAFi.

Following the efforts to understand the molecular basis of drug resistance and to establish combination therapies able to revert acquired resistance, studies have focused on miRNAs and their role in controlling and/or reverting drug resistance. In this context, miRNAs act as oncosuppressors. Figure 1 summarizes the involvement of miRNAs in the pathways active in melanoma.

Only a few studies have regarded the impact of miRNAs on BRAFi/MEKi-based targeted therapies. miR-32, a tumor suppressor miRNA, has recently been demonstrated to suppress the growth of melanoma tumors in preclinical models by inhibiting the expression of the myeloid cell leukemia 1 (MCL-1) gene regulating the RAS-RAF-MEK-ERK and the PI3K-AKT-mTOR pathways. Inhibition of MCL-1 through miR-32 may be an effective anti-melanoma strategy, regardless of the status of NRAS, BRAF or PTEN, as MCL-1 inhibition exhibits synergistic effects with Vemurafenib [21]. Low miR-579-3p expression in BRAF-mutated cells is linked to metastatic melanoma progression; expression levels of miR-579-3p decrease from nevi to stage III/IV melanoma samples and even further in cell lines resistant to BRAF/MEK inhibitors. miR-579-3p is able to simultaneously affect both the BRAF/MAPK and the MDM2/p53 pathways. Indeed, in BRAF-mutated cells, further reduction of miR-579-3p expression leads to additional increases in BRAF and MDM2 levels, causing uncontrolled cell growth, enhanced cell proliferation and migration, and inhibition of apoptosis. This condition contributes to the establishment of resistance to targeted therapy. In this regard, it is important to understand the mechanisms responsible for miR-579-3p expression. This miRNA could be regulated by specific transcription factors whose expression is altered during the development of drug resistance. A possible role could be played by a microphthalmia-associated transcription factor (MITF). miR-579-3p is an intronic miR located in intron 11 of the human gene ZFR (Zink-finger recombinase), and it is probably co-expressed with its host gene. Considering that the ZFR gene is supposed to be a target of MITF because its promoter has MITF-consensus binding sites, it has been speculated that MITF downregulation during the development of drug resistance is responsible for decreased expression of downstream miR-579-3p. Hence, miR-579-3p acts not only as an oncosuppressor but also as a factor contributing to the development of drug resistance [22].

Another potent oncosuppressive miRNA is miR-200c. miR-200c expression is significantly reduced in resistant melanoma cells [23]. miR-200c has been proposed to prevent the establishment of drug resistance in melanoma by targeting Bmi-1, Zeb2, Tubb3, ABCG5, and MDR1, transcriptional repressors that belong to a complex signaling network involved in the epithelial to mesenchymal transition (EMT). Reduction of miR-200c increases Bmi-1 expression, which in turn leads to the activation of the PI3K/AKT and MAPK pathways and the acquisition of features seen in EMT, such as downregulation of E-cadherin and upregulation of N-cadherin, ABCG5, and MDR1 [24]. Low levels of miR-200c have been seen to be correlated with the development of resistance to BRAFi in clinical samples of melanomas and BRAFi-resistant cell lines. Restoration of miR-200c expression or knockdown of Bmi-1 in resistant melanoma cells potentiates the effect of MAPK pathway inhibitory drugs and impairs the establishment of resistance, thus suggesting miR-200c as a potential therapeutic target for overcoming acquired BRAFi resistance [24].

miR-7 expression has been shown to decrease in BRAFi-resistant melanoma cells. Introduction of miR-7 decreases the expression levels of EGFR, IGF-1R, CRAF in vitro as well as in Vemurafenib-resistant cells in melanoma xenograft mice models, which indicates that EGFR, IGF-1R, and CRAF are the target genes of miR-7 that are associated with the acquired resistance to BRAFi. By decreasing the expression levels of EGFR, IGF-1R, CRAF, miR-7 could inhibit the activation of the MAPK and PI3K/AKT pathways and reverse melanoma cell resistance to BRAFi [25].

### 2.2. miRNAs Acting as Oncogenes

miR-21 has been reported to have a potential role in the treatment of CMM. This miRNA is a well-known modulator of cell proliferation, survival, and migration/invasion. Deregulation of miR-21 has been found in many human cancers. In particular, miR-21 expression is frequently upregulated in human cutaneous melanoma and higher levels correlate with advanced tumor stage, degree of invasion and tumor recurrence. Overexpressed miR-21 may function as an oncogene and promote cancer development by negatively regulating genes that control cell differentiation or apoptosis. Many studies have demonstrated that PTEN is a direct target of miR-21 and levels of PTEN, in turn, modulate the activation status of the PI3K/AKT pathway [26] (Figure 1). Antisense-mediated knockdown of miR-21 has been shown to suppress growth, increase apoptosis, and enhance the chemo- or radio-sensitivity of cutaneous melanoma cells. Such observations indicate that targeting miR-21 will be a potential novel strategy for the treatment of CMM. [27].

Some miRNAs, such as miR- 638 and miR-579-3p, have also been described to affect melanoma cell apoptosis alone or in combination with BRAFi treatments [28].

miR-638 is significantly overexpressed in metastatic melanoma. Indeed, miR-638 overexpression enhances the proliferative, migratory, and clonogenic properties of melanoma cells in vitro and their metastatic capacities in vivo. miR-638 induces its pro-tumorigenic and metastatic effects, protecting melanoma cells from apoptosis and autophagy. Knockdown of miR-638 increases TP53INP2 expression and stimulates expression of p53 and p53 downstream target genes, inducing significant apoptosis and autophagy [29].

The other 3 miRNAs, miR-34a, miR-100, and miR-125b, seem to be involved in the control of cell proliferation and apoptosis. They are upregulated in BRAFi-resistant melanoma cell lines and in the biopsy samples from patients treated with Vemurafenib, decreasing sensitivity to BRAFi therapy. In particular, the adaptative cell response to BRAF inhibitors increases expression of RTK and RTK ligands such as the chemokine monocyte chemoattractant protein-1 (CCL-2), which in turn activates the expression of miR-34a, miR-100, and miR-125b. All this leads to an increase in proliferation and resistance to apoptosis. Inhibition of CCL2 and of these miRNAs restores both cell apoptosis and drug efficacy in resistant melanoma cells [30].

In BRAF-mutated patients treated with Vemurafenib, high expression of miR-192 and miR-193b* and low expression of miR-132 has been associated with short time to progression, indicating a poor prognosis [31].

mir-514a has been reported to be involved in the modulation of BRAFi sensitivity in melanoma cells. In particular, miR-514a plays an important role in initiating melanocyte transformation and promoting melanoma growth by regulating the tumor suppressor NF1 gene. miR-514a overexpression in melanoma cell lines inhibits NF1 expression, which correlates with increased survival of BRAFV600E cells treated with Vemurafenib [32]. Both NF1 direct silencing with siRNA and miR-514a upregulation lead to decreased NF1 levels and thus considerably reduce drug sensitivity in the short term in vitro cell proliferation assays [32].

### 2.3. miRNA-Based Therapeutic Approaches

Several studies have highlighted the potential of targeting miRNAs and, therefore, it is likely that many new RNA-based therapeutics will be developed within the next few years [33].

Various strategies have been explored to utilize the role of miRNA to develop anticancer therapeutics:

(a) miRNA inhibition therapy with anti-miRNA oligomers, which are 17–22 nucleotides long, single-stranded, chemically modified, competitive inhibitors of miRNAs, causing upregulation of the target mRNA;
(b) Synthetic miRNA mimetic agents used as replacement therapy to replace or substitute lost miRNA;
(c) Small-molecule inhibitors of miRNA (SMIRs) to either inhibit miRNA biogenesis or impede the interaction of miRNA with its target;
(d) miRNAs targeted during transport within the tumor milieu or to distant sites in microvesicles and exosomes [34].

A recent approach to enhance or suppress miRNA function includes delivering synthetic oligoribonucleotides (ORNs) that copy the native miRNA duplex in which high miRNA expression is needed to reproduce single-stranded antisense RNA to use the targeted endogenous miRNA for inhibition studies. Developing modified duplex RNAs that retain their biological activity is a real challenge, and trials to deliver a single-stranded mature miRNA have been unsuccessful [35] probably because of the inability of duplex RNAs to load into the RNA-induced silencing complex. Thus, continuous efforts have been made to improve the stability and cellular uptake of miRNA to be used to treat various diseases.

Despite various studies demonstrating the involvement of miRNAs in melanoma and in specific therapeutic approaches, further systematic research is required to identify miRNAs able to modulate drug resistance. In fact, preclinical studies involving organoids and validation in a large cohort will be key to uncovering the role of miRNA in response to therapies or as molecules to be targeted.

## 3. The Advent of Immune Checkpoint Inhibitors: Do miRNAs Have a Role?

The advent of immunotherapy approaches has marked a step forward in the treatment of cancer. Generally speaking, immunotherapies, more precisely immune checkpoint inhibitors (ICIs), are based on the interaction between tumor cells and the immune microenvironment, in particular the adaptive immune response and the cytotoxic activity of T lymphocytes.

Antibodies against Cytotoxic T-Lymphocyte Antigen 4 (CTLA-4) and Programmed cell Death 1 (PD-1) (Ipilimumab and Nivolumab/Pembrolizumab, respectively) were the first agents to be approved by the FDA. In 1996, the antitumor activity of the CTLA-4 blockade was shown in mice and in 2010, the exceptional results of the phase III MDX010-20 study led to the registration of Ipilimumab for advanced melanoma.

In 2014, Nivolumab was approved by the FDA for metastatic melanoma treatment. Based on the results of clinical trials, anti-PD1 antibodies have shown the highest efficacy in melanoma. The CheckMate-066 trial demonstrated improved survival rates and progression-free survival in 210 naïve patients with unresectable/metastatic melanoma treated with Nivolumab [5]. The KEYNOTE-006 trial reported the same results and a durable response after discontinuation of immunotherapy [6,36].

Notwithstanding the remarkable clinical results, no biomarkers have yet been approved for ICI response in melanoma. Elevated serum lactate dehydrogenase (LDH), which is an independent predictor of survival in melanoma according to the American Joint Committee on Cancer (AJCC) guidelines [37], has been shown to be related to a worse outcome in patients treated with Ipilimumab [38] and Pembrolizumab [39,40]. KEYNOTE-006 trial found that LDH is not correlated to the duration of the remission period, even if it could be considered a useful biomarker for monitoring disease, as described by Diem et al. [41]. PD-L1 expression measured by immunohistochemistry was evaluated as a biomarker but the antibodies to be used for detection and cut-off are still under investigation. The results of the Checkmate-066 could not show that PD-L1 could be considered as a predictive biomarker for ICI response because the subset of responding patients included both PDL-1 positive and negative subjects [5]. The introduction of next-generation sequencing approaches has made it possible to explore the genome and transcriptome for every type of tumor. Whole exome sequencing (WES) and RNAseq has been extensively used in several melanoma studies. WES measures the tumor mutational burden (TMB), which is very high in melanoma. Snyder et al. [42] were the first to demonstrate, in a cohort of 64 patients treated with anti-CTLA4, that TMB is associated with a good response to ICIs and they identified a neoantigen signature associated with clinical benefit. Neoantigens derive from somatic mutations, which give rise to mutated proteins presented by antigen-presenting cells (APC). However, Van Allen et al. [43] were not able to confirm the predictive role of the neoantigen signature seen in the study performed by Snyder and colleagues and concluded that the recurrence of neoantigens was a rare event. In another study, Roh et al. [44] did not confirm the association of TMB with an ICI response, even if they found copy number loss was associated with poor outcomes. Several groups have focused on transcriptome analysis to set up a gene expression signature to stratify patients but to date, they have proved discordant [43,45,46]. Identifying biomarkers for ICI response in melanoma represents an unmet clinical need. miRNAs have been evaluated as potential biomarkers, even if, as stated by Dragomir et al. [47], molecular networks could be more helpful in the definition of miRNAs able to influence immune response and thus be considered as biomarkers. Immune checkpoint genes, as any biological process, could be regulated both directly by miRNAs and indirectly through proteins, which in turn can be influenced by miRNAs. Recently, an integrative analysis of deregulated mRNAs, miRNAs, and long noncoding RNAs (lncRNAs) between metastatic and non-metastatic samples included in the TCGA repository led to the definition of a competing endogenous RNA network [48]. Survival analysis of the 3 types of RNAs included in the network demonstrated that 6 lncRNAs, 7 mRNAs, and 5 miRNAs were associated with the prognosis of metastatic melanoma. Such a bioinformatic approach and the use of publicly available data could be applied to define a network of RNAs able to predict response to ICIs.

To date, results regarding miRNAs and ICI responses are sparse, but studies could gain momentum by using the single-cell RNAseq approach to distinguish between the expression of tumor cells and immune components.

miR-29 a/b/c isoforms were studied in 2009 and miR-29a was the first miRNA that was demonstrated to bind to B7-H3 3′UTR and whose expression was found to be inversely related to that of an immune checkpoint inhibitor, in different tumors, B7-H3 [49]. In cutaneous melanoma, an inverse relationship between miR-29c and B7-H3 has been found [50]. Recently, B7-H3 has been proposed as an immune inhibitory protein due to its role in the inhibition of T cell proliferation [51,52]. Promising results have been reported in a clinical trial treating recurrent metastatic neuroblastoma patients with an anti-B7-H3 monoclonal antibody, 8H9 [53]. The metastatic potential of B7-H3 has been dissected in melanoma cells through a silencing technique, and results showed a decrease in the proteins involved in metastasization, namely matrix MMP-2, STAT3 and Il-8 [54].

Moreover, the expression of miR-155 is stimulated by IRF4, which is overexpressed in CD8 T cells from Murine lymphocytic choriomeningitis virus chronic infection [55]. Thus, Martinez-Usatorre and colleagues concluded that miR-155 could be considered a marker of responsiveness of CD8 T cells, as further demonstrated by its upregulation after PD1 blockade [56].

The relationship between T-cell exhaustion and miRNA expression has been investigated. The definition of T-cell exhaustion is controversial [57], but, generally, exhausted T cells are not functional in cancer [58] and overexpress inhibitory receptors like TIM3, PD-1, and BTLA [59,60,61]. Microarray-based profiling was performed in PD1+ and PD1- CD4 T cells sorted from lymph nodes and spleen of tumor-bearing mice. The significantly decreased expression of miR-28, miR-150, and miR-151-5p in PD1+ CD4 T cells was validated but miR-28, in particular, was found to silence PD1 through 3′UTR binding [62]. Moreover, exhausted T cells showed a reduced secretion of IL-2, TNF-α and IFN-γ and the use of miR-28 mimics was able to restore their secretion [62].

PD-L1 overexpressing melanoma have been shown to be resistant BRAFi and MEKi. Audrito et al. [63] demonstrated that miR-17-5p binds to PD-L1, suggesting its role in the resistance to targeted therapies.

Martinez-Usatorre et al. [56] studied the role of miR-155, which had proved to be regulated during CD8 T cells differentiation in previous investigations [64]. They measured miR-155 expression in CD8 T cells isolated from tumor-infiltrating lymph nodes and tumor tissue samples from melanoma patients and murine models. miR-155 expression in CD8 T cells was found to depend on antigen stimulation [56]. Previous investigations had shown that TCR (T Cell Receptor) is responsible for NF-kB and AP1 activation that in turn, upregulates miR-155 expression [65,66]. Moreover, the expression of miR-155 is stimulated by IRF4, which is overexpressed in CD8 T cells from Murine lymphocytic choriomeningitis virus chronic infection [55]. Thus, Martinez-Usatorre and colleagues concluded that miR-155 could be considered a marker of responsiveness of CD8 T cells, as further demonstrated by its upregulation after PD1 blockade [56].

## 4. miRNAs and the Interplay with Tumor Microenvironment

The tumor microenvironment (TME) is a complex network composed of soluble factors, extracellular matrix, and several types of cells, including endothelial cells, fibroblasts, and immune cells. The interplay between tumor cells and its TME ensures the maintenance of proliferation and, eventually, sprouting of malignancies. Given the significant role of the TME, bioinformatic algorithms based on RNAseq and microarrays have been developed for the deconvolution of bulk gene expression data to infer TME composition computationally (for example, through CIBERSORT [67]).

Several factors, including oxidative stress, pH variation, and acidosis, regulate the dynamics of TME. HIF1α and HIF2α are key players in the response to hypoxic conditions. The survival of melanoma cells in a low oxygen environment has been found to be linked to a low expression of miR-211, which functions as a metabolic regulator through its interaction with PDK4. Downregulation of miR-211 in melanoma cells drive PDK4 overexpression, leading to a decrease in pyruvate dehydrogenase and, in turn, in oxidative phosphorylation [68]. Neo-angiogenesis is another of the mechanisms known to be stimulated by tumor cells [69] to favor proliferation in oxygen/nutrient low environments. ApoE, a suppressor of angiogenesis and cell invasion [70], has been shown to be targeted by miR-1908, miR-199a-5p, and miR-199a-3p in melanoma, thus highlighting the possibility to target them [70,71]. In the previous section, we focused on the relationship between miRNAs and checkpoint inhibitors. miRNAs are able to regulate more generally both innate and adaptive immunity. miR-210 in melanoma inhibits the lysis of tumor cells by T cells, by regulating TNF-α, IL-6, and IFN-β [72]. M1 polarization of macrophages is regulated by miR-29a, and miR-21 is overexpressed in the blood of melanoma patients, targeting COL4A2, SPARC, and TIMP3 [73].Through the interaction between the NKG2D receptor and its ligand NKG2DL, Natural Killer (NK) cells are able to kill tumor cells but this ability is impaired by miR-34a/c and miR-449a/miR-449c [74]. In addition, Myeloid-Derived Suppressor Cells (MDSCs), whose role in cancer is still under evaluation, while its immune suppressive role is well-known [75], could be directly/indirectly regulated by miRNAs. miR-155 is able to induce MDSCs recruitment in TME [76], inhibiting SOCS1. Moreover, MDSC function is influenced by miR-494, whose expression is induced by TGFβ1 [77].

The EMT constitutes one of the hallmarks of cancer because of its role in resistance to treatments due to the acquisition of invasiveness. The crosstalk with the EMT (e.g., metabolic modification, stroma/immune cells, growth factor, and hypoxia) is responsible for the switch of tumor cells [78]. Melanoma cells are not epithelial but EMT markers have been identified, which are negatively correlated with the state of differentiation of melanocytes [79,80]. miR-205-5p, miR-542-3p, miR-9, and miR-31 target different pathways and regulate EMT components [81,82,83,84,85].

## 5. Circulating MicroRNA Biomarkers in Melanoma

Although therapeutic options for melanoma have substantially changed in the past few years, the development of non-invasive methods for monitoring disease progression or treatment resistance continues to be a major challenge. Recently, the use of liquid biopsy results has proved to be useful as a source of non-invasive biomarkers that provide the entire genetic panorama of the tumoral landscape, allowing for earlier intervention and melanoma therapeutic decisions. Liquid biopsy is defined as the detection of circulating tumor cells (CTCs) or tumor-derived nucleic acids such as tumor DNA (ctDNA), mRNA, or miRNA that are released into circulation by cancer cells [86]. It is not an invasive method thus it can be used repetitively, unlike classic biopsy.

In the past decade, circulating miRNAs have emerged as powerful novel tools for the diagnosis and monitoring of patients with melanoma [87]. Most miRNAs are found within the cells, however, low levels are also detectable in the extracellular space including the bloodstream where miRNAs, referred to as circulating miRNAs, are attached to lipoproteins, proteins, or loaded inside exosomes. Exosomes are little vesicles derived from endosomes and are released from cells by fusion of the multivesicular endosome with the plasma membrane. Like other extracellular vesicles, exosomes contain proteins, RNA, miRNA, DNA, and lipids [88] that are delivered to the intercellular environment playing a pivotal role in cell–cell communication. Loading of circulating miRNAs into exosomes prevents degradation by serum and plasma RNases [89,90]. As such, circulating miRNAs are very stable under the harsh conditions of the blood [91], a feature that points to their potential use as easily accessible markers to help clinicians monitor cutaneous melanoma progression and treatment response [92].Since 2010, numerous efforts have been made to prove that circulating miRNAs are useful as melanoma diagnostic biomarkers. Table 1 summarizes the circulating miRNAs described in the literature as potential biomarkers in melanoma. Leidinger et al. [93] were the first researchers to use a microarray-based approach to screen ~866 human miRNAs in blood cells and the qRT-PCR technique to validate a 16-miRNA signature able to distinguish melanoma patients from healthy controls.

More recently, Fogli et al. [94] studied 30 patients with different stages of melanoma showing upregulation of plasma miR-15b-5p, miR-149-3p, miR-150-5p, and downregulation of miR-193a-3p and miR-524-5p in patients with melanoma compared to healthy subjects, suggesting that these 5 miRNAs may be new potential biomarkers in human cutaneous melanoma. Following their investigations, Li P. et al. [95] indicated that circulating miR-221-3p was a useful biomarker for staging. Lower serum levels of this miRNA were observed in stage I–II patients than in stage III–IV patients. A recent study by Stark et al. [96] measured the expression of a panel of 17 miRNAs (MELmiR-17) in melanoma tissue and serum samples from 255 melanoma patients and 130 controls. The authors also indicated that a seven-miRNA panel (MELmiR-7) made up of miR-16, miR-211-5p, miR-4487, miR-4706, miR-4731, miR-509-3p, and miR-509-5p was a useful tool to predict melanoma progression and recurrence, with a good relevance also in melanoma diagnosis and prognosis.

In addition to studies on the identification of circulating miRNAs with diagnostic power, several investigations regarded miRNAs able to distinguish drug responders from non-responders and predict melanoma recurrence and progression [97]. Kanemaru and co-workers [98] indicated circulating miR-221-3p as a biomarker for melanoma exhibiting with significantly different levels between stage I/IV melanoma patients and healthy controls. They described how miR-221-3p levels decreased after surgical removal of the primary tumor and increased upon disease recurrence, suggesting that circulating miRNA-221-3p could have a role as a new tumor marker.

Fleming et al. [99] identified a serum miRNA signature, including miR-150, miR-15b, miR-425, and miR-30d, which distinguished recurrent from non-recurrent cases and stratified patients into groups at high and low risk of recurrence. Friedman et al. [100] screened 355 miRNAs in sera from 80 melanoma patients at primary diagnosis and identified a 5-miRNA signature, which comprised miR-150, miR-15b, miR-199a-5p, miR-33a, and miR-424 and was able to distinguish patients with a high risk of recurrence from those with a low risk. In agreement with the study of Fleming et al. [99], upregulation of miR-150-5p and downregulation of miR-15b-5p were observed in the serum of melanoma patients at high risk of recurrence. Friedman et al. [100] also reported a signature of 5 miRNAs able to classify melanoma patients into high and low recurrence risk. A longitudinal evaluation of circulating miRNA expression in pre- and post-recurrence serum samples of 17 melanoma stage II patients highlighted a significant increase in the expression levels of miR-103a-3p and miR-221-3p at the time of primary diagnosis and upon recurrence.

Similarly, Tian et al. [101] suggested that miR-206 could be used as a potential prognostic and predictive biomarker, demonstrating that its levels in the serum of melanoma patients were associated with disease progression, poor prognosis, and response to treatment. Margue et al. [102] identified a set of 8 miRNAs that were profoundly deregulated in late-stage melanoma patients. Among them, miR-193b-3p and miR-720 discriminated melanoma patients from healthy controls and non-metastatic from metastatic melanoma patient groups. The first study that identified a circulating miRNA panel useful to detect the presence of metastasis in melanoma patients was performed by Shiiyama and co-workers [103]. It suggested that serum miR-9-5p, miR-145-5p, miR-150-5p, miR-155-5p and miR-205-5p could be used as prognostic biomarkers to discriminate between primary and metastatic melanoma patients. Some years later, Van Laar et al. [104] identified a 38-miRNA signature (MEL38) able to discriminate melanoma from normal plasma samples and an 18-miRNA signature (MEL18) able to distinguish between non-metastatic (stage I/II) and metastatic (stage III/IV) melanoma patients.

### Exo-miRNAs

Tumor-derived exosomes also play a role in epigenetic regulation. They contain various enzymes that are involved in the synthesis and regulation of miRNAs, the most abundant RNAs in exosomes (exo-miRNA) [105]. In the past few years, miRNA profiles of tumor exosomes were found to be correlated with tumor burden or disease risk and thus, the detection of exo-miRNA has been indicated as a promising, non-invasive method for cancer diagnosis and a new tool for drug delivery [106]. Using miRNA profiling, Rappa et al. [107] revealed 49 different miRNAs with higher concentrations in metastatic-melanoma derived microvesicles, named prom1-exo, than in parental cells. Among these deregulated miRNAs, 20 proved to have a specific cancer-related function. Later, Alegre and co-workers [108] examined the exosome-associated miRNA pool of melanoma patients and controls. Significantly lower levels of miR-125b were observed in the serum exosomes of patients with advanced melanoma than in those of disease-free patients and healthy controls. However, no significant difference was observed between the miRNAs from the whole serum of melanoma patients and from that of healthy controls. More recently, Tengda L et al. [109] detected the levels of 5 miRNAs, namely miRNA-532-5p, miRNA-106b, miRNA-200c, miRNA-199a-5p, and miRNA-210, in serum exosomes isolated from 30 melanoma patients and 30 healthy individuals. Serum exo-miRNA-532-5p and exo-miRNA-106b proved to have the potential to be used as biomarkers for the diagnosis and monitoring of melanoma in a clinical setting. The authors also developed and subsequently validated an exo-miRNA panel in 95 serum samples from melanoma patients and healthy controls and concluded that it was a powerful diagnostic tool to distinguish patients with metastasis from those without metastasis, stage I-II patients from stage IV-V patients, and patients who had received Pembrolizumab treatment from those who were untreated.

Several studies have suggested using circulating miRNAs for melanoma staging and recurrence prediction. However, the lack of reproducibility among the results reported by different research groups constitutes a substantial obstacle for the future use of circulating miRNAs in clinical practice. To date, there have been very few multi-center studies, and cohorts have often been insufficiently powered. The lack of standardized analytical methods and pre-analytical procedures, together with the use of different technological platforms and statistical methodologies, has contributed to these discrepancies. Adequate standardization of methods is required before circulating miRNAs can be used in clinical trials to investigate their potential as diagnostic and prognostic biomarkers for melanoma management.

## 6. Conclusions

This review clearly highlights the urgent need to identify novel biomarkers of response/resistance to therapies for melanoma treatment. miRNAs regulate the expression of genes involved in the pathways affected by targeted therapies and immune checkpoint inhibitors. miRNAs have been found to stratify patients in terms of diagnosis and prognosis. The interplay with TME and exo-miRNAs modulates the states of the cytotypes interacting with melanoma cells, favoring proliferation, and invasion.

## Figures and Tables

**Table 1:** Circulating miRNAs with a diagnostic and predictive role in melanoma.

Main Deregulated miRNA	Body Fluid Type	Role	Detection Method	Reference
miR-186, let-7d*, miR-18a*, miR-145, miR-99a, miR-664, miR-501-5p, miR-378*, miR-29c*, miR-1280, miR-365, miR-1249, miR-328, miR-422a, miR-30d, miR-17*	Blood	Prognostic	microarray/qRT-PCR	[93]
miR-15b-5p, miR-149-3p, miR-150-5p, miR-193a-3p, miR-524-5p	Plasma	Prognostic	qRT-PCR	[94]
miR-221-3p	Serum	Prognostic	qRT-PCR	[95,98]
MELmiR-17 panel (hsa-miR-211, miR-508-3p, miR-514a, miR-4731, miR-146a, miR-509-3p, miR-506-3p, miR-509-5p, miR-508-5p, miR-4487, miR-16, miR-204, miR-513c, miR-513b, miR-145, miR-363-3p, miR-4706) and MELmiR-7 panel (miR-16, miR-211-5p, miR-4487, miR-4706, miR-4731, miR-509-3p, miR-509-5p)	FFPE tissue and serum	Prognostic and Predictive	qRT-PCR	[96]
miR-150, miR-15b, miR-425, miR-30d	Serum	Prognostic and Predictive	qRT-PCR	[99]
miR-150, miR -15b, miR -199a-5p, miR-33a, miR-424	Serum	Prognostic and Predictive	qRT-PCR	[100]
miR-206	Serum	Prognostic and Predictive	qRT-PCR	[101]
miRNome including miR-193b-3p and miR-720	Serum, whole blood samples, melanoma tissue, primary melanocyte and keratinocyte cell lines	Prognostic and Predictive	qPCR arrays	[102]
miR-9, miR-145, miR-150, miR-155, miR-203, and miR-205	Serum	Prognostic	qRT-PCR	[103]
38-miRNA signature (MEL38) and 18-miRNA signature (MEL18)	Plasma	Prognostic and Predictive	microarray	[104]
miR-216b, miR-889, miR-4307, miR-4272, miR-203, miR-4289, miR-3149, miR-203, miR-3145, miR-1911, miR-513a-3p, miR-3916, miR-886-3p, miR-1182, miR-3613-5p, let-7i, miR-3132, miR-3914, miR-3618, miR-1307, miR-3614-3p, miR-3160, miR-519c-3p, miR-3153, miR-4278, miR-3646, miR-3926, miR-515-5p, miR-3169, miR-10a, miR-140-5p, miR-3148, miR-4271, miR-627, miR-548d-3p, miR-3613-3p, miR-481, miR-571, miR-4274, miR-4277, miR-3686, miR-3074, miR-95, miR-590-3p, miR-525-5p, miR-548g, miR-365, miR-525-3p, miR-320d	exosomes	Prognostic	microarray	[107]
miR-16, miR-125b	serum exosomes	Prognostic	qRT-PCR	[108]
miR-532-5p, miR-106b, miR-200c, miR-199a-5p, miR-210	serum exosomes	Prognostic and Predictive	qRT-PCR	[109]
